# Evaluation and Management of Septic Arthritis and its Mimics in the Emergency Department

**DOI:** 10.5811/westjem.2018.10.40974

**Published:** 2018-12-06

**Authors:** Brit Long, Alex Koyfman, Michael Gottlieb

**Affiliations:** * Brooke Army Medical Center, Department of Emergency Medicine, Houston, Texas; † The University of Texas Southwestern Medical Center, Department of Emergency Medicine, Dallas, Texas; ‡ Rush University Medical Center, Department of Emergency Medicine, Chicago, Illinois

## Abstract

Septic arthritis is a dangerous medical condition associated with significant morbidity and mortality. However, the differential diagnosis can be broad with conditions that mimic this disease and require different evaluation and treatment. This narrative review presents the emergency medicine evaluation and management, as well as important medical conditions that may mimic this disease. Septic arthritis commonly presents with monoarticular joint pain with erythema, warmth, swelling, and pain on palpation and movement. Fever is present in many patients, though most are low grade. Blood testing and imaging may assist with the diagnosis, but the gold standard is joint aspiration. Management includes intravenous antibiotics and orthopedic surgery consult for operative management vs. serial aspirations. Clinicians should consider mimics, such as abscess, avascular necrosis, cellulitis, crystal-induced arthropathies, Lyme disease, malignancy, osteomyelitis, reactive arthritis, rheumatoid arthritis, and transient synovitis. While monoarticular arthritis can be due to septic arthritis, other medical and surgical conditions present similarly and require different management. It is essential for the emergency clinician to be aware how to diagnose and treat these mimics.

## INTRODUCTION

Monoarticular arthritis is a common presentation to the emergency department (ED) and major cause of disability in the United States. Monoarticular arthritis has a wide range of potential etiologies, ranging from benign to life-threatening. One of the most concerning causes in a patient with monoarticular arthritis is septic arthritis. The prevalence of septic arthritis among ED patients with monoarticular arthritis varies significantly between studies; however, an incidence of 4–60 cases per 100,000 population per year is suggested in the literature.^1^–^6^ Based on the literature, higher rates of septic arthritis are present in immunocompromised patients and those with prosthetic joints, where disease incidence increases to 70 cases per 100,000 patients annually.^7^–^13^ Septic arthritis possesses a bimodal incidence, with peaks in both childhood and adults over the age of 55 years.^4^–^9^

Septic arthritis consists of a bacterial infection of the joint space that is associated with rapid joint destruction within days if not adequately treated. Mortality rates can be significant, ranging from 3–25%.^3^,^5^–^7^ Despite the severity of illness, septic arthritis may be subtle, with many patients lacking the classic signs, symptoms, or laboratory findings.^8^–^10^ There are also a large number of conditions that may mimic septic arthritis, further confounding the diagnosis.

## METHODS

We searched PubMed and Google Scholar for articles using the keywords “septic arthritis,” “monoarthritis,” “synovial fluid,” “diagnosis,” “treatment,” and “emergency.” Restricting the literature search to studies published in English, we found an initial 258 articles. We reviewed all relevant articles and decided by consensus which studies to include for the narrative review, focusing on articles investigating ED patients, studies evaluating synovial fluid results, and studies investigating septic arthritis diagnosis or management. A total of 133 articles were selected for inclusion in this review. We did not conduct a systematic review or meta-analysis, but rather a narrative review evaluating the emergency medicine investigation and management of septic arthritis and its mimics.

## DISCUSSION

Septic arthritis typically affects one joint but may be polyarticular in up to 20% of cases (most commonly in immunocompromised patients).^10^,^14^,^15^ The most frequently affected joint is the knee, followed by the hip, shoulder, and elbow.^8^–^11^ Septic arthritis results from bacteremia in 70% of cases due to the absence of a protective basement membrane within the joint lining.^8^–^11^,^15^–^29^ This provides easy passage of bacteria into the synovial fluid. Other causes include direct inoculation from trauma or a medical procedure and contiguous spread from osteomyelitis, an abscess, cellulitis, or septic bursitis.^8^–^11^,^15^–^18^

### Organisms

The majority of cases are due to Gram-positive organisms (e.g., *Staphylococcus aureus*), with approximately 15% being due to Gram-negative organisms (Table 1).^15^–^25^ The incidence of methicillin-resistant *S. aureus* (MRSA)-related septic arthritis is increasing.^20^ *Neisseria gonorrhoeae* is another common cause in younger adults; these patients can present with migratory polyarthritis, pustular rash, urethritis, and tenosynovitis.^8^–^11^,^15^,^17^ Polymicrobial infections (e.g., *Pantoea agglomerans* and *Nocardia asteroides*) typically occur after penetrating trauma, such as bite wounds, or with organic foreign material.^6^–^10^,^18^–^25^ Small breaks in the skin and mucous membranes provide entry points for Gram-positive bacteria, while Gram-negative infections result from injection drug use, gastrointestinal sources, or urinary tract mucosal injury.^8^–^11^,^15^–^28^ Once bacteria are present within the normally sterile synovial fluid, the body sends immune cells to the site of infection.^8^–^11^,^15^,^26^,^27^ The combination of bacteria within the joint capsule, the host inflammatory response, and tissue ischemia can result in significant joint damage.^10^,^26^,^27^

### History and Examination

Obtaining an accurate history and assessment of risk factors can provide important clues to the diagnosis. A careful evaluation for risk factors can significantly change a provider’s pretest probability of septic arthritis.^8^,^9^ Table 2 provides sensitivity, specificity, positive likelihood ratio (+LR), and negative likelihood ratio (−LR) for various history and examination findings.^8^ Of note, this table combines values from several meta-analyses.^8^,^9^ Several of the findings were not available for pooling of data due to heterogeneity and unreliable methodology of included studies. The most common risk factor is preexisting joint disease or damage; however, this is present in less than half of patients with septic arthritis.^6^–^8^,^10^ Other risk factors are typically related to the route of the infection, including hematogenous (e.g., injection drug use), direct inoculation (e.g., trauma or recent procedure), or contiguous spread (e.g., abscess).^8^–^10^,^18^

While each risk factor in isolation has only a modest impact on the likelihood of septic arthritis, the overall risk rises as the number of risk factors increases.^8^–^10^ Many patients with septic arthritis possess several risk factors.^6^–^11^,^15^,^16^ For example, patients with rheumatoid arthritis are at an increased risk for septic arthritis due to joint damage, poor skin condition, and immunosuppression.^26^,^29^ Rheumatoid arthritis complicated by septic arthritis is associated with poor outcomes including high morbidity and mortality.^10^,^29^,^30^ Interestingly, one study found that approximately 22% of all patients with culture-proven septic arthritis had no associated risk factors or underlying joint disease.^30^ This can be partly explained due to septic arthritis from *N. gonorrhoeae* in young patients with otherwise normal joints, though most cases of septic arthritis were due to *S. aureus*.^30^

Patients traditionally present with a constellation of signs and symptoms including joint pain, tenderness to palpation, swelling, erythema, warmth, and painful or limited range of motion.^8^,^9^,^17^ The most common symptom is joint pain, which is found in 85% of patients.^8^,^9^ Joint swelling occurs in 78% of cases,^8^,^9^ while joint tenderness has been suggested to be 100% sensitive.^6^,^7^,^15^,^17^ Fever ≥ 39^o^C occurs in up to 58% of patients, and the absence of fever should not be relied upon to exclude the diagnosis; however, up to 90% of patients have been shown to have a low-grade fever (≥ 37.5^o^C).^8^,^9^ Joint pain that is sudden in onset is more suggestive of intrinsic joint pathology, such as septic arthritis.^8^–^10^,^17^,^18^ A joint with painful and limited active and passive range of motion is suggestive of intra-articular infection.^8^,^9^

### Laboratory Testing

Serum blood tests are inadequate to rule out septic arthritis. Synovial fluid is the gold standard test for making the diagnosis of septic arthritis. While a complete blood cell count, C-reactive protein (CRP), and erythrocyte sedimentation rate (ESR) are often obtained, the results of these tests will not sufficiently lower the post-test probability to influence the decision to obtain synovial fluid.^8^–^110^,^17^,^18^ The serum white blood cell (WBC) count may be elevated above 10 × 10^9^/liters (L), but the sensitivity ranges from 42–90% with +LR of only 1.4 (95% confidence interval [CI] [1.1–1.8]).^8^,^9^,^31^–^36^ The sensitivity of ESR differs based upon the specific cut-off value that is selected, with a sensitivity of 66% for 15 mm/hr to > than 90% for 30 mm/hr.^7^–^10^,^30^,^35^–^37^ One meta-analysis suggests a +LR of 1.3 (95% CI [1.1–1.8]) for ESR > 30 mm/hr.^9^ CRP > 10 mg/L also has a sensitivity approaching 90%; however, a level of 100 mg/L has a poor +LR of 1.6 (95% CI [1.1–2.5]).^8^,^9^,^35^ While procalcitonin demonstrates promise, at this time it requires further study before routine use.^8^,^10^,^17^,^18^,^38^,^39^ Blood cultures should be obtained in patients with septic arthritis, as they can help identify the source if the synovial fluid culture is negative. Blood cultures will be positive in over one-third of all patients, and 14% of patients with negative synovial fluid cultures will have positive blood cultures.^6^,^10^,^15^,^17^,^18^

### Imaging

Radiographs are typically obtained of the affected joint and may demonstrate soft tissue swelling or a joint effusion.^10^,^40^,^41^ Later stages of septic arthritis may reveal chronic bony changes and calcium deposits.^10^ Advanced imaging, including computed tomography and magnetic resonance imaging, possesses greater sensitivity and specificity than plain radiographs, though it is of low utility for the acute diagnosis.^10^,^40^–^42^ Ultrasound may provide assistance in determining the presence of intra-articular effusion and locating the site of optimal aspiration.^10^,^26^,^43^,^44^

### Synovial Fluid

Synovial fluid is the gold standard for excluding septic arthritis in patients with high clinical suspicion. Results of the aspiration also assist with determining the etiology of joint effusion (Table 3). However, some of these findings may overlap between categories.^8^,^17^,^18^,^45^ The numbers from this table have been obtained from several meta-analyses and are provided here in one location.

A synovial white blood cell count (sWBC) ≥ 50 × 10^9^/L is concerning for septic arthritis (Table 3).^8^,^9^,^17^,^18^ Moreover, the likelihood of septic arthritis increases as the sWBC rises, with levels ≥ 100 × 10^9^/L demonstrating an aggregate +LR of 13.2 (95% CI [3.6–51.1]).^8^–^10^ While the sWBC values can affect the likelihood of septic arthritis, it is important to consider that the patient’s immune status may affect these findings, resulting in low sWBC counts in patients with significant immunocompromised status.^8^,^9^,^45^ A sWBC ≥ 50 × 10^9^/L (or 50,000 cells/mm^3^) may also be found in several other inflammatory conditions (e.g., gout, pseudogout).^8^–^10^,^17^,^18^,^32^ Additionally, nearly half of patients with culture-proven septic arthritis may have sWBC counts ≤ 28,000 cells/mm^3^, even in cases due to *S. aureus,* with *N. gonorrhoeae* accounting for 5% of all cases.^8^–^10^,^17^,^18^,^32^ Synovial polymorphonuclear cells (sPMN) can also be significantly elevated in cases of septic arthritis.^8^,^9^,^15^ Unfortunately, this test does not significantly alter probability of septic arthritis, with a +LR of 2.7 (95% CI [2.1–3.5]) when the sPMN is > 90% and a −LR of 0.34 when the sPMN is < 90%.^8^,^9^

Other diagnostic assessments include synovial Gram stain, culture, protein, lactate dehydrogenase (LDH), glucose, and lactate.^8^–^10^,^15^,^17^,^18^ Synovial culture is the single most important test and should be ordered on all patients from whom synovial fluid is collected. Synovial fluid will demonstrate growth in approximately 80% of all cases of nongonococcal septic arthritis.^8^–^10^ The remaining 20% of negative cultures may demonstrate no growth for a variety of reasons including small number of bacteria present within the joint space, obtaining a sample after initiation of antibiotics, mistaken diagnosis of septic arthritis, poor sampling technique, or poor plating technique.^8^–^10^,^17^,^18^,^45^ To decrease the likelihood of false negative synovial cultures, larger amounts of synovial fluid should be collected and placed in blood culture bottles. Synovial Gram stain sensitivity ranges from 29–65% in cases of Gram-positive septic arthritis; however, this decreases to 40–50% in Gram-negative cases and 25% in gonococcal cases.^15^–^18^,^45^–^53^

Synovial protein and glucose do not significantly change the likelihood of septic arthritis.^8^,^9^ One study found that a synovial lactic dehydrogenase less than 250 U/L may exclude the diagnosis of septic arthritis, but further studies are needed.^8^,^53^ The presence of crystals does not rule out septic arthritis.^8^,^10^,^17^,^18^,^45^,^54^ Synovial lactate has been suggested to have the best diagnostic accuracy of all synovial fluid markers in septic arthritis. Levels above 10 mmol/L demonstrate a +LR > 20.^8^,^51^,^55^–^57^ Of note, it is important that the laboratory be able to differentiate D-lactate, produced by bacteria, from L-lactate, produced by humans.^8^,^57^ Therefore, this may not be feasible at all institutions.

### Management

Rapid diagnosis and treatment reduce the risk of significant morbidity and mortality.^10^,^17^,^18^,^58^,^59^ Risk factors associated with increased risk of joint destruction include age > 65 years, diabetes, and beta-hemolytic streptococci infection, while risk factors for mortality include age > 65 years, confusion at time of initial presentation, and polyarticular involvement.^30^,^59^–^61^ Components of management include early recognition and treatment, with 1) joint aspiration, 2) antibiotics, and 3) orthopedic surgery consultation for possible operative management.^10^,^17^,^18^,^58^,^59^

Due to the potential for rapid joint destruction, broad-spectrum antibiotics are often needed.^17^,^18^,^58^,^59^ In patients with strong concern for septic arthritis or in those who are critically ill, both Gram-negative and MRSA coverage is recommended with a combination of cefepime or an antipseudomonal beta-lactam agent and vancomycin, respectively.^17^,^18^,^58^,^59^ If the patient is allergic to vancomycin, daptomycin, clindamycin, or linezolid may be utilized instead.^17^,^18^,^58^,^59^ Once the specific organism is determined, antibiotic therapy should be narrowed. There is currently no role for intra-articular antibiotics or intra-articular corticosteroids for these patients in the ED setting.^10^,^58^

While many patients may be managed with antibiotics alone, it is important to involve orthopedic surgery, as some patients may require arthroscopy, serial arthrocentesis, or arthrotomy in addition to the antibiotics.^10^,^17^,^18^,^58^,^59^ Arthrocentesis removes bacteria and toxins, decompresses the joint space, and improves blood flow, which may improve recovery.^10^,^17^,^18^,^58^,^59^ Arthrocentesis is typically repeated on a daily basis until cultures are negative and effusions resolve.^10^,^17^,^18^,^58^,^59^ In cases that fail to respond to serial arthrocentesis, soft tissue infections that extend outside of the joint or involvement of the hip joint, surgical drainage is often indicated.^1^,^58^,^59^ Septic arthritis involving the shoulder may be managed with surgical or radiologically-guided techniques.^10^,^58^–^60^ Some joints, such as the sternoclavicular joint, do not respond well to antibiotics alone.^58^–^64^ In these cases, cardiothoracic surgical consultation is recommended.^58^–^64^

### Joint Aspiration

Most joint aspirations are within the purview of the emergency physician.^10^,^58^,^59^ While it is traditionally recommended to avoid aspirating through a site with overlying cellulitis, one recent review suggested there was no harm from aspirating through cellulitis, with the only direct definitive contraindication an underlying abscess.^65^ Additionally, anticoagulation is a relative contraindication, but should be weighed against the much higher risk associated with missing a case of septic arthritis.^66^ Prosthetic joints should be discussed with orthopedic surgery prior to aspiration.^67^ If unable to obtain fluid on the initial aspiration, several techniques may be used to increase the likelihood of success. Using a larger gauge needle and a smaller syringe can improve the ability to obtain fluid by generating a greater pressure difference.^68^ Additionally, compression of the contralateral side of the joint with gentle rotation of the needle while aspirating will be of benefit.^68^ Finally, ultrasound should be considered for arthrocentesis, as it locates the area with maximal fluid, while avoiding vascular structures and tendons.

### Special Considerations

#### Gout

Gout can predispose patients to septic arthritis due to chronic joint damage.^8^,^10^,^54^,^69^ Patients with a first instance of an erythematous, swollen, painful joint and those with atypical presentations of their usual gout should undergo joint aspiration. Joint fluid in gout traditionally demonstrates uric acid, or calcium pyrophosphate crystals in pseudogout; however, it is important to note that these crystals do not exclude concomitant septic arthritis, as the pathologies may coexist in up to 5% of cases.^54^,^69^ Patients with gout and septic arthritis often demonstrate sWBC counts > 50 × 10^9^/L;^54^,^70^ however, up to 10% of patients may demonstrate sWBC < 6 × 10^9^/L.^70^ Patients with concern for possible septic arthritis should undergo joint aspiration, antibiotics, orthopedic consultation, and admission.^17^,^18^,^69^,^70^

#### Human Immunodeficiency Virus (HIV)

Patients with human immunodeficiency virus and acquired immunodeficiency syndrome are predisposed to a variety of orthopedic conditions, including infections and vascular infarctions due to a chronic immunocompromised and inflammatory state.^8^,^10^,^71^–^73^ In this population, septic arthritis is most commonly associated with MRSA, though tuberculosis and fungal species have also been identified.^71^–^73^ Patients may not be able to produce a normal immune response to septic arthritis, resulting in lower sWBC levels.^71^–^73^ Patients with either new or chronic joint pain with effusion should undergo aspiration given the high risk of opportunistic infections.

#### Prosthetic Joint

Prosthetic joint infection (PJI) occurs most commonly within the first two years after surgery, with a rate of 1–2% for hip and knee arthroplasties and 1% with shoulder arthroplasty.^67^,^74^–^77^ Unlike native joints, prosthetic joints do not contain cartilage and are not at risk of cartilage destruction.^67^,^77^ Acute infections (i.e., < six weeks from operation) should receive urgent antibiotics to preserve the prosthesis, while more chronic infections (i.e., > six weeks from operation) may be treated with less urgency.^67^ Chronic infection is more common than acute postoperative and acute hematogenous infection in these patients.^78^,^79^ Risk factors for PJI include longer procedural time, postoperative wound drainage, obesity, malnutrition, diabetes, anticoagulants, tobacco use, heavy alcohol use, poor hygiene, prior surgery at the same site, and bacterial colonization.^79^–^84^ *S. aureus* is the most common organism, followed by *S. epidermidis* and *Pseudomonas* due to the production of a protective bacterial biofilm.^84^–^86^

Signs and symptoms depend upon the patient’s immune response and whether the infection is acute or chronic.^67^ Acute infections typically present with a new effusion, erythema, and warmth combined with general symptoms of fever and malaise, while chronic infections may present with more subtle signs of pain over time without significant external evidence of infection.^67^,^76^,^87^ Findings may also include an open wound, sinus tract, or abscess.^67^,^76^,^88^,^89^ If there is concern for a PJI, the physician should obtain serum laboratory testing (i.e., WBC, ESR, CRP) and perform a joint fluid aspiration in consultation with the patient’s orthopedic surgeon.^67^,^88^–^90^ Cultures from a draining wound are not recommended due to risk of skin flora contamination.^67^,^76^ Diagnostic criteria are shown in Table 4.^67^,^76^

Importantly, the specific thresholds for septic arthritis differ compared to native joints. For acute PJI, thresholds of sWBC 10 × 10^9^/L and sPMN > 90% are recommended.^90^–^92^ For chronic PJI, sWBC 3 × 10^9^/L and sPMN > 80% are recommended.^74^,^75^,^88^,^89^ One publication recommended joint aspiration for a CRP > 100 mg/L for acute infection.^67^ Revision surgery and antibiotics are usually required. However, compared with native joint infections, these are typically not needed emergently.^67^,^76^ If patients present with fever and an acute onset of symptoms, blood cultures should be obtained and antibiotics administered in the ED.^67^,^76^ Otherwise, antibiotics may be withheld until the case is discussed with the orthopedic surgeon.^67^,^76^

#### Hemophilia

Hemarthrosis is a common presentation among patients with hemophilia A and B.^93^–^97^ This is a hallmark of more severe hemophilia and is associated with chronic disability and reduced quality of life.^93^–^96^ Hemarthrosis can result in chronic joint damage and increases the risk of septic arthritis at a rate of 15–40 times that of the general population.^93^–^96^ Patients with hemophilia who have joint pain, swelling, or erythema should be asked about prior hemarthroses, factor levels, prophylactic medications, and recent factor administration. In most patients, joint aspiration should be avoided in the setting of hemarthrosis.^97^,^98^ However, if the patient presents with severe pain, fever, joint erythema, or swelling in the absence of trauma and septic arthritis is suspected, aspiration of synovial fluid is important.^93^–^96^ Aspiration of hemarthrosis may improve pain and rehabilitation in patients with rapid intra-articular accumulation of blood, although this is controversial.^97^,^98^ Before conducting aspiration of suspected hemarthrosis, emergency physicians should discuss the aspiration with hematology and orthopedics, specifically addressing possible factor replacement prior to joint aspiration.^97^,^98^

#### Mimics

A significant number of conditions may mimic the presentation of septic arthritis, creating difficulty in diagnosis. Knowledge of these conditions and their presentation, diagnosis, and management may improve patient outcomes. Table 5 demonstrates these conditions, and Appendix 1 lists these mimics with evaluation and management recommendations.

## CONCLUSION

Septic arthritis is a potentially deadly condition that unfortunately does not always present classically. The red, hot, swollen joint mandates consideration of septic arthritis. No physical examination finding can rule out the condition, and serum blood tests should not be used to exclude septic arthritis. Diagnostic aspiration is required, with the sample sent for synovial WBC, Gram stain, culture, and lactate. Synovial lactate and culture are the best laboratory tests, as some patients can present with normal synovial WBC and Gram stain. Management requires orthopedic surgery consultation and antibiotics. There are a significant number of mimics of septic arthritis, including abscess, cellulitis, gout, rheumatoid arthritis, osteomyelitis, malignancy, Lyme disease, and avascular necrosis. A focused history and examination, along with dedicated diagnostic evaluation, can assist in differentiating these conditions.

## Supplementary Information



## Figures and Tables

**Table 1:** Common organisms causing septic arthritis.^6^–^11^,^15^–^26^

Bacteria (frequency)	Clinical characteristics
Staphylococci (56%)	
Methicillin-sensitive *Staphylococcus aureus* (42%)	All: skin breakdown, cellulitis over the site (46% of cases), prosthetic joint, recent operation on joint, damaged joint All: high mortality (7–18%) and joint function loss (27–46%)
Methicillin-resistant *Staphylococcus aureus* (10–50%)	
Coagulase-negative staphylococci (3%)	
Streptococci (16%)	
*Streptococcus viridans* (1%)	All: splenic dysfunction, post splenectomy, diabetes, cirrhosis All: associated with high frequency of bacteremia (66%) and polyarticular disease (32%) All: high mortality (19%), but good functional outcomes in those that survive
*Streptococcus pneumoniae* (1%)	
Unspecified/other streptococci (14%)	
Gram-negative rods (15%)	
*Pseudomonas aeruginosa* (6%)	All: Immunocompromised status, gastrointestinal disorder or infection, injection drug use, elderly Enteric Gram-negative rods: Urinary tract infection found in 50% of patients All: 5% mortality
*Escherichia coli* (3%)	
Proteus species (1%)	
Klebsiella species (1%)	
Others (4%)	
Other (12%)	
Polymicrobial (5%)	All: immunocompromised status, travel or residence in an endemic area, gastrointestinal disorder or infection Neisseria: increases with high-risk sexual activity; 75% occur in women, 72% are polyarticular, 32% have urinary symptoms, recovered from joint fluid in < 50% of cases Tuberculosis: indolent course with gradually progressive joint pain and swelling, symptoms often occur for > 1 year before the diagnosis; only 50% of patients have chest radiograph with active tuberculosis Brucella: more common in immigrants to the United States, typically occurs in regions with unvaccinated livestock and unpasteurized dairy; 54% have sacroiliac joint involvement
Anaerobes (0.6%)	
*Mycobacterium tuberculosis* (1.8%)	
*Neisseria gonorrhoeae* (1.2%)	
Brucella (1–11%)	
Miscellaneous (4%)	

**Table 2:** History and examination findings in septic arthritis.*^8^,^9^

Finding	Sensitivity	Specificity	−LR (95% CI)	+LR (95% CI)
History				
Age > 80 years	18.9	94.6	0.86 (0.70–0.96)	3.5 (1.7–6.4)
Rheumatoid arthritis	67.6	72.5	0.45 (0.27–0.67)	2.5 (1.9–2.9)
Diabetes	10.8	96.0	0.93 (0.79–1.0)	2.7 (1.1–6.2)
Joint surgery (< 3 months)	24.0	96.5	0.78 (0.63–0.90)	6.9 (3.7–11.6)
Hip or knee prosthesis	35.1	88.6	0.73 (0.55–0.88)	3.1 (1.9–4.5)
Skin infection, no prosthesis	32.4	88.4	0.76 (0.58–0.91)	2.8 (1.7–4.2)
Skin infection and prosthesis	24.3	98.4	0.77 (0.62–0.88)	15.0 (8.0–26.0)
HIV	75.0	38.8	0.64 (0.23–1.37)	1.2 (0.76–1.5)
Joint pain	85.0	-	-	-
New joint swelling	77.0	-	-	-
Rigors	16.0–21.0	-	-	-
Fever, subjective	44.0–97.0	-	-	-
Diaphoresis	31.0	-	-	-
Physical examination				
Limited motion	92.0	-	-	-
Pain with motion	100	-	-	-
Pain with axial loading	36.0	-	-	-
Tender to palpation	68.0–100	-	-	-
Swelling	45.0–92.0	-	-	-
Joint effusion	92.0	-	-	-
Erythema	13.0–64.0	-	-	-
Increased heat on palpation	18.0–92.0	-	-	-
Fever > 37.5^0^C	34.0–90.0	-	-	-

*−LR,* negative likelihood ratio; *+LR,* positive likelihood ratio; *CI,* confidence interval; *HIV,* human immunodeficiency virus.

* Remaining numbers represented by hyphens could not be calculated due to heterogeneity and unreliable methodology.^8^,^9^

**Table 3:** Categories of synovial fluid findings in monoarticular arthritis.

Synovial fluid measure	Normal fluid	Noninflammatory	Hemorrhagic	Inflammatory	Septic
Color	Clear	Yellow	Red	Yellow	Yellow/green
Clarity	Transparent	Transparent	Bloody	Translucent-opaque	Opaque
Viscosity	High	High	Variable	Low	Variable
White blood cells	< 2 × 10^9^/L	< 2 × 10^9^/L	< 2 × 10^9^/L	2–100 × 10^9^/L	10–100 × 10^9^/L
Percentage of PMNs	< 25%	< 25%	50–75%	> 50%	> 75–80%
Culture result	Negative	Negative	Negative	Negative	Usually positive

Synovial result		+LR (95% CI)		−LR (95% CI)	
sWBC > 100 × 109/L		13.2 (3.6–51.1)		0.83 (0.80–0.89)	
sWBC > 50 × 109/L		4.7 (2.5–8.5)		0.52 (0.38–0.72)	
sWBC 25–50 × 109/L		3.2 (2.3–4.4)		0.35 (0.23–0.50)	
sPMN > 90%		2.7 (2.1–3.5)		0.51 (0.39–0.65)	
sLactate > 10 mmol/L		> 20*		0.14–0.45*	

*PMNs,* polymorphonuclear neutrophil; *sWBC,* synovial white blood cell count; *sPMN,* synovial polymorphonuclear cell count; *sLactate,* synovial lactate; *CI,* confidence interval; *+LR,* positive likelihood ratio;* −LR,* negative likelihood ratio; *L,* liter.

* Unable to pool results to obtain accurate 95% confidence intervals.

**Table 4:** Musculoskeletal Infection Society definition of periprosthetic joint infection.^67^,^76^

Two positive periprosthetic cultures with phenotypically-identified organisms Or A sinus tract communicating with the joint Or Three of the following minor criteria: Elevated CRP and ESR Elevated sWBC or positive leukocyte esterase strip Elevated synovial neutrophil percentage Positive histologic analysis of periprosthetic tissue A single positive culture result

*CRP,* C-reactive protein; *ESR,* erythrocyte sedimentation rate; *sWBC,* synovial white blood cell count.

**Table 5:** Septic arthritis mimics.

Abscess
Avascular necrosis
Cellulitis
Crystal-induced arthropathy
Lyme disease
Malignancy
Osteomyelitis
Reactive arthritis
Rheumatoid arthritis
Transient synovitis
