# Supplementary material for: Evaluation and Management of Septic Arthritis and its Mimics in the Emergency Department
Source: West J Emerg Med. 2018 Dec 6;20(2):331–41. doi: 10.5811/westjem.2018.10.40974 (PMC6404712; doi:10.5811/westjem.2018.10.40974)
Supplement: Supplementary file 1 [file wjem-20-331-s001.doc]

**Appendix 1. Details of Septic Arthritis Mimics**

| **Condition** | **Presentation** | **Diagnosis and Management** |
| --- | --- | --- |
| Abscess^99-104^ | -Over 3 million ED visits for abscesses every year.  -Occurs most commonly in the 19- to 45-year-old age group, but no significant gender differences.  -MRSA is the most common bacteriologic cause.  -Patients present with a swollen, erythematous, fluctuant, painful mass under their skin, commonly legs or arms. | -Laboratory testing is usually non-diagnostic.  -Ultrasound has been demonstrated to increase both the sensitivity and specificity when compared with clinical examination.  -Ultrasound is 96% sensitive and 83% specific.  -Treatment consists of incision and drainage; needle aspiration is not recommended.  -Newer data suggests that antibiotics improve cure rates, though must be weighed against potential adverse events. |
| Avascular Necrosis^105-107^ | -20,000 to 30,000 new cases annually, representing 10% of all total hip arthroplasties in the United States.  -Typically affects young, healthy patients between 20 and 40 years of age.  -Most common affected location is the hip.  -Etiology is suspected to be due to vascular occlusion.  -Risk factors include: femoral head or neck fracture, hip dislocation, radiation, SCFE, sickle cell disease, myeloproliferative disorders, chronic corticosteroid use, excessive alcohol use, smoking, autoimmune disorders, hemoglobinopathies, and coagulopathies.  -Patients may present with minimal symptoms initially or referred pain to the buttock or knee.  -Patients often have limited range of motion at the hip with pain on internal rotation. | -Initial diagnosis is often made on x-ray.  -Lesions are classified using the Ficat and Arlet or Steinberg University of Pennsylvania staging systems.  -MRI is 99% sensitive and specific, which can identify subtle, early lesions.  -Treatment involves reversing the suspected etiology with dietary and pharmacologic interventions, with surgery reserved for patients with more advanced disease or refractory pain. |
| Cellulitis^108-111^ | -Over 2 million ED visits annually.  -Dermal and subcutaneous bacterial infection, most commonly caused by Group A streptococci (*Streptococcus pyogenes*), followed by *Staphylococcus aureus*.  -Patients typically present with focal areas of increased pain, warmth, erythema, and swelling.  -Red streaking may be noted if there is associated lymphangitis.  -The lower extremities are the most commonly affected area.  -Fever is present in 22.5-77.3% of patients. | -Laboratory testing is usually non-diagnostic.  -Blood cultures are generally not indicated and have a high rate of false positives.  -Ultrasound or computer tomography may be used to identify abscesses or other deep space infections.  -Mild cases can be treated with antistreptococcal antimicrobial agents (eg, cephalexin, dicloxacillin, penicillin VK, or amoxicillin/clavulanate). The addition of TMP-SMX for MRSA coverage has not been demonstrated to improve cure rates.  -Severe cases may be treated with intravenous cefazolin, ceftriaxone, penicillin G, clindamycin, vancomycin, or linezolid. |
| Crystal-induced arthropathy^112-116^ | -Crystal-induced arthropathies affect 4% of all adults in the United States.  -Gout is caused by uric acid crystals, while pseudogout is caused by calcium pyrophosphate dihydrate crystals.  -Increased age, male sex, alcohol ingestion, red meat intake, and sugar-sweetened beverage consumption are associated with increased risk of gout flares.  -Patients present with an erythematous, swollen, and tender joint with decreased range of motion.  -The great toe is most commonly affected in gout, while the ankles and knees are most commonly affected in pseudogout. | -The diagnosis is made by analysis of synovial fluid from the affected joint.  -Negatively birefringent, needle-shaped crystals are pathognomonic of gout, while positively birefringent, rhomboid-shaped crystals are seen in pseudogout.  -The acute treatment is focused on reducing inflammation using non-steroidal anti-inflammatory drugs (NSAIDs), steroids, and colchicine.  -Long-term therapy includes dietary changes and initiation of a xanthine oxidase inhibitor (eg, allopurinol). |
| Lyme Disease^117-119^ | -The most common tick-borne illness.  -Incidence is highest among children 5-10 years of age.  -Cases have been reported in every state but is most common in the East Coast and Midwest regions.  -Caused by *Borrelia burgdorferi* transmitted by the *Ixodes scapularis* deer tick.  -Early stages present with a red macule or papule that expands to form an annular erythematous lesion (ie, erythema migrans).  -Late stages present with monoarticular arthritis, with the knee being the most frequently affected location. Less common findings include neurologic manifestations (eg, polyneuropathy or encephalopathy). | -Diagnosis is made clinically, based upon history and examination findings  -Only 50-70% of patients recall a tick bite  -Serologic testing may be sent, but patients should be treated empirically prior to the results.  -Patients should be treated with doxycycline or amoxicillin for early or mild disease.  -Patients with Lyme meningitis should receive intravenous ceftriaxone  -NSAIDs may provide symptomatic benefit.  -Some patients may develop a Jarisch-Herxheimer reaction after receiving the antibiotics, but this is not an indication to stop antibiotic therapy. |
| Malignancy^120,121^ | -Osteosarcoma is the most common primary malignancy of the bone.  -Patients aged 10-14 years have the highest incidence; males are affected more commonly than females.  -Lesions occur most commonly at the metaphyseal region of long bones but can also occur in the skull or axial skeleton.  -Patients typically present with progressively increasing pain and swelling of a specific bony area. | -Initial diagnosis is often made on x-ray.  -Magnetic resonance imaging may be valuable for more subtle cases.  -Patients should be referred to an orthopedic surgeon for further evaluation and management. |
| Osteomyelitis^122-125^ | -Osteomyelitis is an acute or chronic infection of bone caused by either direct inoculation (eg, surgery, trauma) or hematogenous spread (eg, bacteremia, endocarditis).  -The most common etiologic agent is *Staphylococcus aureus*, but there are numerous other bacteria that have been associated with this infection.  -Patients with recent trauma, surgery, diabetes, or immunocompromise are at increased risk of infection.  -Patients present with progressively worsening pain, swelling, and erythema of a specific bone or joint. | -Diagnosis may be made clinically if there is exposed bone or a persistent sinus tract.  -Inflammatory markers (eg, C-reactive protein, erythrocyte sedimentation rate) and blood cultures may assist with diagnosis.  -Radiographs may demonstrate the infection with advanced disease, but bone scintigraphy and magnetic resonance imaging are more accurate for early infections.  -Computed tomography is only 67% sensitive and 50% specific. Therefore, this is not recommended for evaluating osteomyelitis.  -Treatment should be targeted toward the specific organism. Penicillin, nafcillin, or cefazolin may be initiated empirically if there is low suspicion for MRSA. In areas with increased MRSA prevalence, vancomycin is recommended. |
| Reactive Arthritis^126-128^ | -Reactive arthritis is an inflammatory arthritis caused by a culture-proven infection at another site.  -Reactive arthritis has been associated with genitourinary infections (ie, *Chlamydia trachomatis*), gastrointestinal infections (ie, *Campylobacter jejuni, Clostridium difficile, Escherichia coli, Salmonella, Shigella, Yersinia*), and respiratory infections (ie, *Chlamydophila pneumoniae, Mycoplasma pneumoniae*).  -Patients with the HLA-B27 phenotype are at significantly increased risk.  -Patients typically present with the symptoms of the initial associated infection, followed by progressively worsening pain and erythema of > 1 joint. The lower extremities are affected most commonly. | -The diagnosis is often made clinically based upon the initial infection and associated oligoarthritis.  -Diagnosis can be confirmed by obtaining confirmation of the associated bacteria from the infected location (eg, genitourinary polymerase chain reaction, stool culture).  -Inflammatory markers and imaging are of low diagnostic yield.  -Synovial fluid often has < 50,000 white blood cells per high-powered field and negative cultures.  -Treatment should be targeted at the underlying primary infection. |
| Rheumatoid Arthritis^129-131^ | -Rheumatoid arthritis is the most common inflammatory arthritis with a lifetime prevalence of 1% worldwide.  -Onset can occur at any age but presents most commonly in patients aged 30-50 years.  -Risk factors include older age, female sex, smoking, and family history of the disease.  -Patients typically present with pain and stiffness in multiple joints, associated with morning stiffness lasting greater than one hour. Bone swelling may also be present.  -The distal upper extremity joints (eg, wrists, metacarpals, proximal interphalangeal joints) are the most frequently affected area. | -Diagnosis may be determined using the American College of Rheumatology/European League Against Rheumatism Classification Criteria, which include joint involvement, serology, inflammatory markers, and duration of symptoms.  -Inflammatory markers (eg, C-reactive protein, erythrocyte sedimentation rate) may be helpful and are part of the diagnostic criteria.  -Autoimmune studies can also facilitate the diagnosis. Rheumatoid factor is sensitive, but not specific. Anti-citrullinated protein antibody is highly specific for the disease.  -Treatment is focused on pain control with NSAIDs and referral to a rheumatologist who can initiate disease modifying antirheumatic drugs (DMARDs). |
| Transient Synovitis^132,133^ | -Transient synovitis is a self-limited condition of unknown etiology.  -Most patients are 3-8 years of age, with a 2:1 male predominance.  -There is often an antecedent upper respiratory infection.  -Patients can present with hip pain, limp, refusal to bear weight, or decreased range of motion.  -Fever is uncommon, and patients are typically well-appearing. | -Patients typically have a normal white blood cell count, C-reactive protein, and erythrocyte sedimentation rate.  -Kocher’s criteria should be utilized for risk stratification (Non-weight-bearing on affected side, [Erythrocyte sedimentation rate](https://en.wikipedia.org/wiki/Erythrocyte_sedimentation_rate) > 40, Fever > 38.5 °C, [White blood cell count](https://en.wikipedia.org/wiki/Complete_blood_count) > 12,000).  -An x-ray should be obtained to evaluated for fracture or other diseases (eg, Legg-Calvé-Perthes disease, SCFE, or bony lesions).  -Ultrasound can be utilized to identify an effusion and assist with arthrocentesis, if indicated.  -Treatment is supportive, with NSAIDs. |

*Abbreviations: SCFE, slipped capital femora epiphysis; TMP-SMX, trimethoprim-sulfamethoxazole*
